# GH Replacement in the Elderly: Is It Worth It?

**DOI:** 10.3389/fendo.2021.680579

**Published:** 2021-06-15

**Authors:** Silvia Ricci Bitti, Marta Franco, Manuela Albertelli, Federico Gatto, Lara Vera, Diego Ferone, Mara Boschetti

**Affiliations:** ^1^ Endocrinology Unit, Department of Internal Medicine and Medical Specialties, School of Medical and Pharmaceutical Sciences, University of Genova, Genova, Italy; ^2^ Endocrinology Unit, IRCCS Policlinico San Martino, Genova, Italy

**Keywords:** growth hormone, growth hormone deficiency (GDH), growth hormone replacement therapy, IGF-1, elderly, GHD diagnosis

## Abstract

Growth hormone (GH), once the age of linear growth is completed, continues to play a fundamental role for the human body. In adulthood, GH contributes to regulate muscle, cardiovascular and bone metabolism. The same happens in old age, although there is less data on the effect of GH in the elderly. Regardless the age of onset, a reduced quality of life (QoL), an increased cardiovascular risk and an accelerated age-related decline in physical strength have been demonstrated in the elderly with GH deficiency (EGHD). In adults with GH deficiency (AGHD), recent studies suggest a role of GH replacement therapy (GHrt) in improving lean/fat mass ratio, blood pressure, lipid profile, bone metabolism and QoL. Despite these recent studies, there is still a lack of randomized controlled trials proving these positive effects in EGHD. Moreover, the lack of a long-term positive outcome on mortality, and the cost of GHrt could often impact on treatment decision-making and lead to postpone or avoid the prescription. The aim of this mini-review is to summarize the available data on GHrt in EGHD, in order to highlight its weaknesses and strengths and to provide directions to clinicians that will help in the management of this specific set of patients.

## Introduction

The role of growth hormone (GH), which performs its most important functions through its peripheral mediator, the insulin-like growth factor (IGF-1), is certainly of primary importance in growth. In adult life, the pituitary production of GH physiologically decreases and its circulating levels are progressively reduced; however, the integrity of the GH/IGF-1 axis continues to ensure the maintenance of homeostasis of many organs and systems (1). Adult GH deficiency (AGHD) is a specific condition, diagnosed when GH levels in adults are pathologically reduced (2). It could be the continuation of a childhood-onset GH deficiency or begin in adulthood. AGHD commonly results from pituitary tumours, from the treatments of these disorders or traumatic brain injury (3–5). Apart from the physiological age-related decrease in GH and IGF-1 during life span, elderly patients may suffer from GHD (EGHD) (6). To date, several studies have shown that AGHD patients present physical deficiencies such as an increased cardiovascular risk and bone fragility, unfavourable fat/lean mass ratio, reduced muscle strength (2, 7–9), as well as psychological deficiencies, such as impaired quality of life (QoL) and social alienation (10, 11). It has been proved that GHrt results in stable improvement in these alterations (9, 12–14) and that it is safe in both the short and long term (3, 15, 16). The majority of scientific societies do not share a specific opinion on the identification of EGHD cases for which treatment is required, as there are no precise criteria for deciding whether to start therapy or when and if a previously initiated GHrt should be discontinued (17) once old age is reached (6, 16–18).

The aim of this review is to critically analyse the available peer reviewed papers on EGHD and the disease management in the elderly population. The definition of elderly population has undoubtedly changed over the years. Nonetheless, the majority of the analysed studies identify over-65 patients as elderly.

## Diagnosis of Ghd in the Elderly: When To Suspect it and How To Carry it Out

The diagnosis of GHD in adults and in the elderly must be achieved through the use of standard stimulating tests, except for those patients in whom GHD arises from a non-modifiable structural brain defect, already producing partial hypopituitarism (coexistence of minimum 3 pituitary axes deficit) and low serum IGF-1 (< -2.0 SDS). The need to rely on a provocative test is based on the evidence that the simple measurement of the IGF-1 levels do not distinguish between normal and GHD subjects; in fact, a low IGF-1 is a reliable diagnostic indicator of GHD in the presence of hypopituitarism, but a normal IGF-1 does not rule out GHD (17–19). Current guidelines recommend to firstly determine the probability of an impaired pituitary function and to use further diagnostic investigation in those patients with other pituitary deficient axes especially if clinical signs such as dyslipidaemia, central obesity and loss of muscle mass are present. One or two positive stimulus tests are required to formulate the diagnosis of GHD, depending on the pre-test diagnostic suspicion (16, 18). It is recommended that the decision to carry out the diagnostic protocol for EGHD be corroborated by a strong suspicion to avoid false positive results, given the higher incidence of side effects of the GHrt in old age (11). At the same time, the symptoms and signs of GHD are often non-specific (asthenia, fatigue, reduced muscle strength, increased visceral fat, dyslipidaemia, osteoporosis). Therefore, especially in the elderly, formulating a clinical suspicion can be difficult and the diagnosis could be misrecognized. We can infer that establishing an accurate diagnosis of GHD in the elderly is challenging and even more so if considering the variability in response and interpretation of stimulus tests available (19, 20). In fact, these tests lack age-adjusted cut-offs, despite the well-known physiological decrease of GH and IGF-1 with age (21, 22).

The latest guidelines do not provide suggestions on the most appropriate test to diagnose EGHD and no dedicated studies have been performed to define it. Moreover, two of the most widely used tests for the diagnosis of GHD - the insulin tolerance test (ITT) and the glucagon stimulation test (GST) - are generally avoided in the elderly, due to their potential detrimental effect on patients with multiple comorbidities (23, 24). The GHrh plus arginine test seems to have the best accuracy/safety ratio in the elderly. Indeed, the side effects of this test are negligible, with the only limitation of unequal availability. The GH cut-off point after GHrh plus arginine test to determine AGHD is different, depending on the country and the effect of BMI is not always considered (25). In Italy it is established at 9 µg/l in the normal-weight and at 4.2 µg/l in obese people (BMI > 30 kg/m^2^) (26). In the United States, where GHrh was withdrawn from the market in 2008, the better test for EGHD diagnosis seems to be Macimorelin, which has excellent tolerability and minimal side effects (27), despite several pharmacological interferences (28).

## Recombinant Gh Replacement Therapy (Ghrt) In The Elderly: When, How And Why

The effects of GHrt in AGHD have been widely studied and an improvement in most of the metabolic and psychological abnormalities associated with this condition has been recorded (10). Recent studies have suggested that most beneficial effects of GHrt basically last over the long term (29–32). In EGHD, as well as in AGHD, GHrt should be individually tailored and it is recommended that therapy is started at low doses and up titrated according to the clinical response, side effects, and IGF-1 levels. Periodic monitoring of both benefits and adverse events must be ensured in order to appropriately titrate the dose of therapy. Side effects consist primarily on fluid retention and increase in insulin resistance, typically seen at the beginning of therapy and/or after the increase of the dose. Adverse events are more common in the elderly and, generally, disappear with dose reduction or end of therapy (18, 28, 32).

As in AGHD, the goals of treatment in EGHD are an adequate clinical response, the achievement of IGF-1 levels within the normal range for age and the minimization of side effects (25, 33–35). Based on our clinical experience with EGHD, the treatment goal should be to maintain IGF-1 between -1 and +1 SD, in accordance with the findings of a study by Van Bunderen et al., specifically aimed at comparing different target IGF-1 therapeutics (36). However, clinical practice is not uniform (25, 37, 38) and the therapeutic goal is not univocal: for example, American guidelines suggest a wider range (IGF-1 between -2 and + 2 SD) (16).

In EGHD, initial doses of GHrt of 0.1 mg/day are recommended (34).

Toogood et al. conducted a dose-finding study to identify the minimum effective dose in EGHD, concluding that the majority of patients maintains an IGF-1 adequate level on a dose of 0.33 mg/day (39). In our clinical practice an average dose of 0.2 mg/day is generally sufficient to maintain IGF-1 between the normal range. Standard follow-up interval in treated EGHD is initially 1 or 2 months; the up-titration of GHrt dose is carried out with increments of 0.1 to 0.2 mg/day, based on the clinical response, IGF-1 levels, occurrence of side effects and individual considerations. In AGHD, once the maintenance dose is achieved, follow-up can be deferred to approximately 6 to 12 months. In EGHD, shorter follow-up and smaller dose increments are recommended, especially for those patients with other comorbidities such as diabetes mellitus (40). The parameters to be evaluated during treatment are circulating IGF-1, fasting glucose, glycosylated haemoglobin levels, lipid profile, BMI, waist circumference and waist-to-hip ratio.

It is known that GHrt influence thyroid, glucocorticoid and sex hormone requirements; hence, these hormones should be closely monitored during follow-up (16, 34).

Contraindications to GHrt in EGHD are the same ones identified for AGHD: active neoplasia and active proliferative or severe non-proliferative diabetic retinopathy. GHrt should be initiated with caution in pre-existing type II diabetic patients or with a strong family history of cancer (15, 16, 18) (**Table 1**).

**Table 1:** A brief report about clinical suspicion, diagnosis, GH replacement therapy (GHrt) dose and titrating and follow up in elderly patients with GHD (EGHD).

GROWTH HORMONE DEFICIENCY IN ELDERLY		
Clinical suspicion	**Symptoms and signs:**Asthenia, fatigue, reduced muscle strength, increased visceral fat, dyslipidaemia, osteoporosis	**Hormonal assessment:**IGF-I SDS < -2Other deficient pituitary axes
Diagnosis	**- GHrh + Arginine test-** Macimorelin (if GHrh + Arginine is not available);	**Cut-off GH peak (GHrh + Arginine test):**BMI 25-30 kg/m^2^= <9.0 µg/lBMI >30 kg/m^2^= <4.2 µg/l
Dosage	**Starting dose:** 0,1 mg/die – 0,2 mg/dieincreasing dose 0.1 mg every 1-3 months to achieve IGF-1 level in the normal range for age (we suggest -1<SDS<1)	Daily subcutaneous injection
Follow up	Serum IGF-1, fasting glucose, glycosylated haemoglobin, lipid profile, other pituitary axes, BMI, W/H ratio, waist circumference	Every 1-3 months until the maintenance dose is achieved, then every 6 months
Absolute Contraindications	Active neoplasia, diabetic retinopathy	
Caution	Diabetes Mellitus, family history of cancer	

W/H, waist to hip.

To date, only few randomized placebo controlled trials have assessed the effects of GHrt in EGHD and there are no data on the efficacy and long-term safety in patients above 80 years of age (41). Given the benefits of GHrt in AGHD, and considering the similarity between some signs and symptoms of GHD and of aging, GHrt has been proposed in the past as anti-aging agent in healthy elderly subjects. However, the use of GHrt in this setting appears marginal and its benefits are offset by troublesome side effects (42).

## Cardiovascular Effects

**Figure 1:**
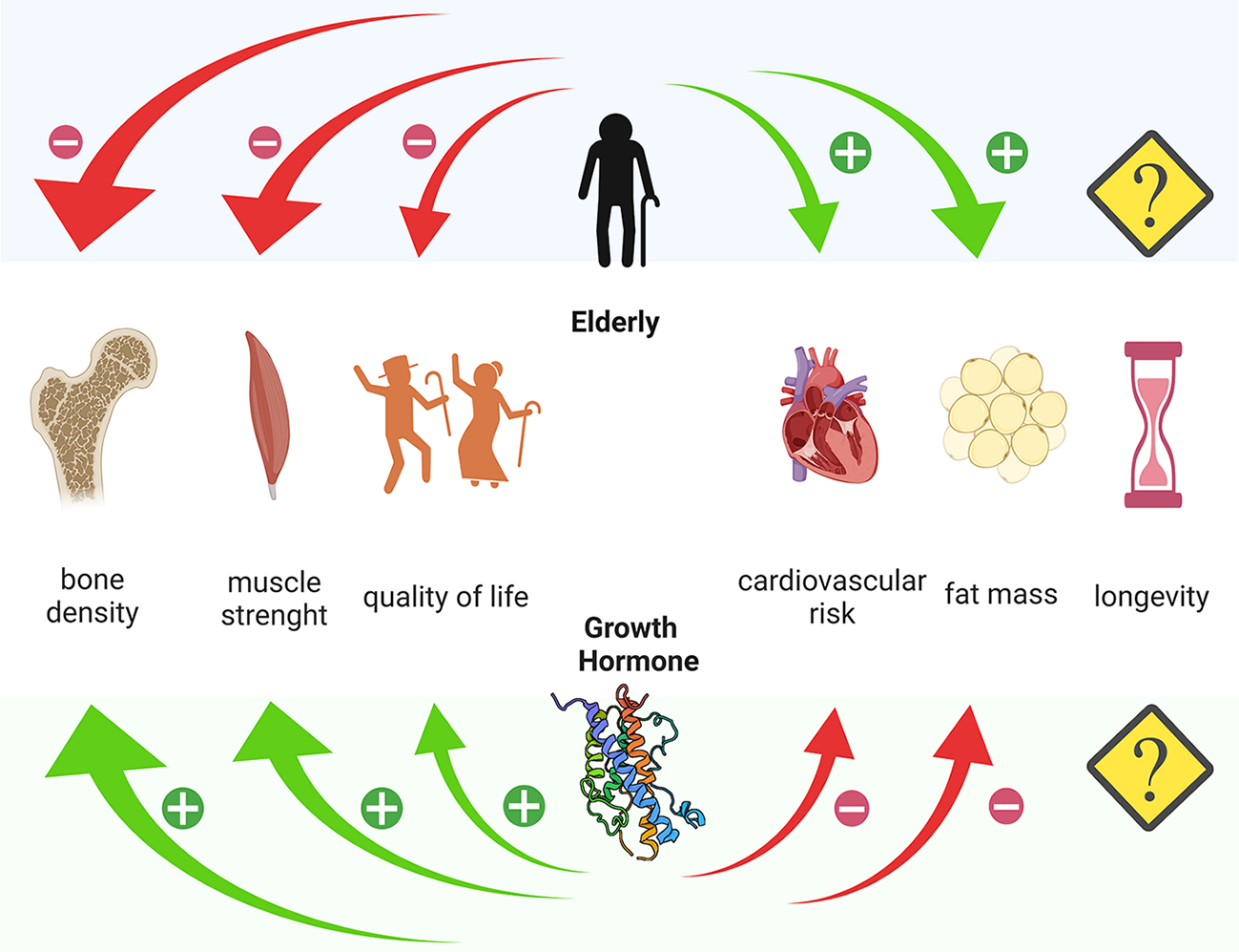
Different action of age and Growth Hormone on bone density, muscle strength, quality of life, cardiovascular risk and fat mass. Created with Biorender.com.

The effects of GHrt on the cardiovascular system in the elderly are the most studied to date. In EGHD patients, attention to cardiovascular risk should be a priority, given the demonstrated increase in cardiovascular morbidity and mortality from this cause in elderly patients and the supposed, although not yet unequivocally defined, increase in cardiovascular risk related to GHD itself (7, 14, 31, 43–45). Some studies show an improvement in HDL/LDL ratio after GHrt of up to 20% (11, 46, 47). In a study comparing treated EGHD and AGHD it was shown that, despite a lower dose of GHrt was used in EGHD, this group surprisingly displayed a more pronounced reduction in waist-to-hip ratio and LDL cholesterol levels (14). In 31 EGHD (age range 60-79 years) Elgzyri et al. demonstrated that GHrt leads to a transient increase in heart rate, an improvement in the resistance to a maximal exercise and an improved LDL/HDL ratio (44). These data suggest the importance to bring IGF-1 levels to the physiological threshold in order to reduce the cardiovascular risk in elderly patients with GHD. There were no clear consistent effects of GHrt on arterial blood pressure, as confirmed by many studies, especially in registries (46). Moreover, the reduction in waist-to-hip ratio after GHrt in EGHD appears controversial (11, 47–51). Considering the few existing studies and their heterogeneity in terms of patients, enrolment criteria and study design, it still remains difficult to establish the power of benefits in terms of clinical implications, such as reduced cardiovascular morbidity and mortality (41) (**Figure 1**).

## Effects on Cognitive Function

A higher incidence of mental disorders, more pronounced mental distress and cognitive dysfunctions are also symptoms of AGHD (10). However, the positive effect of GHrt, in terms of cognitive function, remains doubtful. In healthy elderly subjects, it has been shown that some cognitive aspects correlate inversely with IGF-1 levels (52, 53) and that regional cerebral blood flow, during the performance of memory tasks, increases more in healthy elderly with high circulating IGF-1 than in a group with “low” IGF-1 levels (54).

Sathiavageeswaran et al. in 2007 carried out the first double blind, randomized, placebo-controlled study to establish the effects of GH on cognition in EGHD. It has been found that certain aspects of cognitive function improved over the years in the GHrt group, while the placebo group deteriorated even further. There were no effects in patients without cognitive impairment at baseline. Therefore, the results of this study provide a basis for further investigations in this setting of patients (55).

## Effects on Muscle Strength

Reduced levels of GH and IGF-1 are known to correlate with the grade of impairment of muscle strength (**Figure 1**). Indeed, a recent Chinese cross-sectional study of more than 3200 healthy elderly patients demonstrated how IGF-1 levels negatively correlate with the incidence of sarcopenia in both sexes (56). In AGDH, GHrt significantly improves muscle strength over the years (57). In EGHD, the correlation between GHD and muscle strength is still controversial, due to a very limited number of studies and assessed subjects. Based on the results of a prospective open-label study evaluating the effect of 10 years of GHrt on muscle strength in 24 EGHD (61-74 years), it seems that GHrt does not directly increase muscle strength, but it may reduce its age-related decline (58). Prospective studies in larger populations might be necessary.

## Effects on Body Composition and Bone Metabolism

It is known that in AGHD, the imbalance between fat and lean masses is in favour of the former, (**Figure 1**) and there are some data suggesting an improvement in fat/lean mass ratio after GHrt. In EGHD, the data on this subject are still scarce. Toogood et al. demonstrated that EGHD show significant differences in body fat distribution but the response to treatment was not assessed (50). Moreover, more than one study, demonstrate a positive effect of GHrt on body composition (14, 39, 49), and the data extracted from the large KIMS database also support this evidence (51).

In AGHD, GHrt induces a progressive increase in bone mass and density (29), especially after 5–6 years of treatment (59), but results considerably differ according to age, gender, duration and schedule of treatment, including the dose (60, 61). According to the few studies available, GHrt in EGHD seems to improve bone metabolism (49, 62)**;** however, given the lack of long-term prospective controlled studies, there is no clear evidence of a direct impact on the risk of fracture. This is a crucial point, because sarcopenia is an important risk factor for falls and fractures. Therefore, the maintenance of muscle strength (58) might have beneficial effects on reduction of falls and, indirectly, it could also reduce the fracture risk. We believe that targeted studies are needed to prove this assumption.

## Effects on Quality of Life (Qol)

Reduced QoL in AGHD patients is one of the most consolidated evidence in the literature. GHrt improves QoL in AGHD patients, particularly by increasing energy and stabilizing emotionality (3, 30). Many studies have shown that the improvement in QoL seems more proportional to the degree of the baseline evaluation rather than the changes in IGF-1 levels (28). The assessment of QoL was proposed as a part of the clinical management in GHD patients, complementary to the measurement of surrogate biological markers or other clinical end points. In fact, in the NICE guidance the QoL score questionnaire is mandatory to decide whether to continue the GHrt (63) or not.

In a large recent study (64) including GHD patients older than 50 years old, 4 years of GHrt resulted in beneficial effects in terms of QoL, but no relevant differences were found in GHrt response between early or late initiation of treatment. Li Voon Chong et al. were among the first groups to study QoL in EGHD. They demonstrated that those patients had reduced energy, hypo-mobility, and lack of fulfilment in personal life, became socially isolated and suffered from mental fatigue (65). Remarkable improvements in QoL have been described after 6 months of GHrt and this has long been considered the goal of this therapy in patients with GHD (51). QoL should always be measured using validated questionnaires, such as the QoL-AGHDA (66) and we believe that in EGHD patients, an assessment of QoL, in addition to other clinical parameters, may be a valid determinant of whether to initiate replacement therapy or not.

## Discussion

We can confirm that GHrt in EGHD can contribute to the restoration of a physiological state of health, without inducing significant adverse effects, in particular when the treatment is properly and individually titrated. The main, though few, evidences concern improvements in QoL, cardiovascular risk factors and metabolic features; however, targeted studies on this population are strongly recommended to confirm these results, to adequately test the effectiveness and safety of GHrt in old age and to optimize diagnostic aspects (i.e. to determine peak GH cut-offs stratified by age). It must also be considered that fat mass increases in the elderly and, therefore, correcting the cut-off for weight alone is not always reliable.

We believe it would be important to select suitable patients for treatment, taking into account their health status, comorbidities, life expectancy and on-going medications (67), as well as adherence to chronic treatment (i.e., cognitive status, presence of a caregiver, *etc*.). There are currently several on-going studies with long-acting recombinant GH preparations (68), which use a variety of technologies to prolong the action of GH by deferring administration over time (e.g., weekly). This is intended to improve patient compliance, especially among the elderly, who often take a high number of medications (28).

The evidence available to date suggests, in patients with hypopituitarism, GHD contributes to excess mortality and GHrt contributes to bring mortality rates to those of normal subjects, but a true relationship between mortality reduction and GHrt has not been conclusively established with long-term prospective controlled trials (69). However, it seems unlikely that such studies could be easily conducted, especially in EGHD (11, 49, 69).

We can conclude by stating that the main goal of GHrt in the elderly patient is to improve QoL, prolong independence, and avoid frailty (69, 70). The age-related decline in GH-IGF-1 levels does not justify GHrt supplementation, but patients with established GHD should be considered for treatment, regardless of age, but rather taking into account general conditions, comorbidities, and life expectancy, analysing each case individually (34, 37, 38). We add that considering the costs of GHrt and the mainly long-term effects, the cost/benefit ratio must always be carefully evaluated (38), especially in a population with a reduced life expectancy (71).

In our opinion, the main goal of GHrt in EGHD should be a significant improvement in QoL, which can only be achieved through the development of personalized treatment and careful follow-up. To achieve this, it is necessary to expand studies of GHrt in the elderly population.

## Author Contributions

SR and MB conceived and wrote the manuscript. MF, MA, LV and FG realized the bibliography research. DF supervised the draft. All authors contributed to the article and approved the submitted version.

## Conflict of Interest

The authors declare that the research was conducted in the absence of any commercial or financial relationships that could be construed as a potential conflict of interest.
